# Comparison of the Efficacy and Safety of PARP Inhibitors as a Monotherapy for Platinum-Sensitive Recurrent Ovarian Cancer: A Network Meta-Analysis

**DOI:** 10.3389/fonc.2021.785102

**Published:** 2021-11-24

**Authors:** Hongmei Wang, Meng Wu, Haonan Liu, Hang Zhou, Yang Zhao, Yifan Geng, Bo Jiang, Kai Zhang, Bo Zhang, Zhengxiang Han, Xiuping Du

**Affiliations:** ^1^ Department of Oncology, The Affiliated Hospital of Xuzhou Medical University, Jiangsu, China; ^2^ Department of Hematology, The First People’s Hospital of Lianyungang, Jiangsu, China

**Keywords:** PARP inhibitors, ovarian cancer, monotherapy, maintenance treatment, network meta-analysis

## Abstract

**Background:**

The present COVID-19 pandemic has tended toward normality. To provide convenient, safe, and effective home treatment programs for patients with recurrent ovarian cancer (ROC), the clinical efficacy and safety of poly (ADP-ribose) polymerase inhibitor (PARPi) (including olaparib, niraparib, and rucaparib) monotherapy as a maintenance treatment for platinum-sensitive ROC were systematically evaluated.

**Methods:**

Numerous electronic databases were systematically searched for randomized controlled trials (RCTs) of PARPi maintenance treatment for ROC that were published before June 2021. The primary endpoints were overall survival (OS) and progression-free survival (PFS), and the secondary endpoint was grade 3-4 adverse effects (AEs). After data extraction and the quality evaluation of the included studies, Bayesian network meta-analysis (NMA) was performed using R software. The ability of each treatment was ranked using the surface under the cumulative ranking (SUCRA) curve.

**Results:**

The analysis included five studies and 1390 patients. The NMA results demonstrated that compared with the placebo, olaparib and niraparib exhibited significant benefits in the gBRCA-mutated population, and respectively reduced the risk of death by 31% (HR = 0.69, 95% CI: 0.53-0.90) and 34% (HR = 0.66, 95% CI: 0.44-0.99). Olaparib, niraparib, and rucaparib were all found to be very effective in prolonging PFS in patients with ROC. All three PARPi treatments increased the number of grade 3-4 AEs in patients with ROC as compared with the placebo.

**Conclusions:**

Overall, olaparib and niraparib maintenance treatment can significantly prolong the OS of patients with gBRCA mutations. Furthermore, the three investigated PARPi monotherapy maintenance treatments can prolong PFS regardless of BRCA mutation status. Although the incidence of AEs in the treatment groups was found to be significantly higher than that in the placebo group, the patients in the treatment group tolerated the treatment. Home oral PARPi treatment can balance tumor treatment and pandemic prevention and control, and is the most convenient, safe, and effective home treatment method available against the background of the current COVID-19 pandemic.

**Systematic Review Registration:**

https://inplasy.com/inplasy-2021-6-0033/.

## Introduction

Ovarian cancer is one of the main causes of cancer deaths in women, and is also the most common gynecological cancer worldwide. According to the data of GLOBOCAN 2020, there are 313,959 new cases of ovarian cancer worldwide and 207,252 deaths each year (1). At present, the incidence of ovarian cancer in China is exhibiting an upward trend, ranking the third among malignant tumors of the female reproductive system, and its mortality rate is the highest (2). There is no effective screening strategy for ovarian cancer, and the early symptoms are mostly insidious. Therefore, in many women with ovarian cancer, the disease is already at an advanced stage when it is diagnosed, and the 5-year survival rate is only 15%-25% (3). Surgery and platinum-based chemotherapy have always been the main treatments for ovarian cancer. While patients usually respond well to platinum-based compounds and taxane-based first-line chemotherapy drugs, most will experience relapse and resistance within 12-18 months after the initial treatment (4). At present, the treatment of recurrent ovarian cancer (ROC) has made significant progress, but the overall prognosis remains poor (3, 5).

As the COVID-19 pandemic is spreading internationally, although the large-scale outbreak is under control, the prevention and control tasks remain difficult due to the increase of the floating population caused by work resumption. ACE2, the receptor *via* which COVID-19 invades cells, is highly expressed in a variety of tumors. Genetic abnormalities such as ACE2 mutations and abnormal copy number amplification may occur in tumors, and tumors with high ACE2 expression may be more susceptible to COVID-19. Moreover, tumor patients must repeatedly visit the hospital to undergo surgery, chemotherapy, and radiotherapy, and their resulting low immunity makes them more susceptible to COVID-19 infection than the general population (6, 7). Currently, experts point out that under the premise of pandemic prevention, the principle of the treatment plan for cancer patients should be convenient, safe, and effective to avoid frequent medical visits and reduce the chance of infection (8). Therefore, it is necessary to determine and select more effective and safer treatment strategies to treat ROC, aiming at delaying the recurrence of cancer and improving the overall prognosis of patients while avoiding COVID-19 infection.

Evidence shows that maintenance therapy is effective in extending the remission period of ovarian cancer (9–11). In recent years, molecular targeted drugs, the most important of which include poly (ADP-ribose) polymerase inhibitors (PARPis), have achieved promising results in ovarian cancer-related clinical trials, thereby providing new strategies for the treatment of ovarian cancer. As a class of oral molecular targeted drugs, PARPis, including olaparib, niraparib, and rucaparib, are currently used for the maintenance treatment of platinum-sensitive ROC. These three drugs were respectively approved for the treatment of ROC from December 2014 to July 2017 (12), and are recommended for the treatment of platinum-sensitive ROC by the NCCN Guidelines (13).

Previous studies and published network meta-analysis (NMA) results have confirmed that PARPis have a significant effect in improving the progression-free survival (PFS) of ROC patients (12, 14–17). However, due to the insufficient number of related studies and different inclusion criteria, there has been no comparison of these three PARPi monotherapy maintenance treatments. Overall survival (OS) is the gold standard that can directly reflect clinical efficacy (18, 19). However, because it is affected by subsequent treatment and requires longer follow-up, previous studies have not yet obtained relevant OS data, let alone conducted a specific analysis of OS. Therefore, the long-term efficacy of olaparib, niraparib, and rucaparib as single-agent maintenance treatments of platinum-sensitive ROC lacks favorable evidence. In this study, NMAs were conducted on these three PARPis from the aspects of OS, PFS, and grade 3-4 adverse effects (AEs) to provide more intuitive and effective guidance for clinical medication.

## Materials and Methods

This article was written in strict accordance with the Preferred Reporting Items for Systematic Reviews and Meta-Analyses (PRISMA) (20), and has been registered on the INPLASY website (INPLASY202140014), doi:10.37766/inplasy2021.6.0033.

### Search Strategy and Study Selection

The PubMed, EMBASE, Cochrane Library, and Web of Science databases were systematically searched. The time limit was set from the library construction date to June 2021. The following search terms were used: ovarian cancer, PARP inhibitors (including olaparib, niraparib, rucaparib, etc.), maintenance, and randomized controlled trials; these search terms also included their corresponding subordinate entry terms. In addition, the clinical trial registration website (http://www.clinicaltrials.gov) was searched to obtain more specific information about registered randomized controlled trials (RCTs). When duplicate publications regarding the same clinical trial were found, the most complete and most recently updated study was included.

### Eligibility Criteria

The inclusion criteria of eligible studies were as follows: (I) research design: all articles were prospective phase II or phase III RCTs; (II) research objects: two groups of patients were diagnosed with ROC, primary peritoneal cancer, and fallopian tube cancer *via* pathological examination; (III) intervention: the intervention group was treated with PARPis and the control group was treated with a matched placebo; (IV) outcome: the primary outcomes were overall survival (OS) and progression-free survival (PFS), while the secondary outcome was grade 3-4 adverse effects (AEs) during the maintenance treatment.

In addition, studies were excluded if they met the following criteria: (I) the publication type was a review, a letter, a case report, comments, or an editorial; (II) the test design in the literature was an *in vitro* test, an animal test, or not an RCT test; (III) the research object was newly diagnosed advanced ovarian cancer; (IV) the data were insufficient or unavailable; (V) the included documents did not meet the diagnostic criteria; (VI) non-English publications.

### Data Extraction and Quality Assessment

Two investigators independently reviewed the articles, and disagreements were resolved by discussion and consensus. Using a standardized data collection form, the following information was collected from each study: abbreviation of the study, clinical register, first author’s name, country, participants’ ages, histopathology, BRCA gene mutation status, homologous recombination defect (HRD) status, intervention measures, and outcome indicators.

All eligible studies were evaluated for risk of bias using the Cochrane Risk of Bias tool provided by the Cochrane Intervention System Evaluation Manual (version 5.3.0) (21). The evaluation criteria and content mainly included random allocation, allocation concealment, blinding, incomplete outcome data, selective outcome reporting, and other biases.

### Statistical Analysis

The mtc.network command in the GeMTC package of R3.6.1 software was used to construct the research evidence network diagram. The nodes of the evidence diagram represented different intervention measures, and the lines between the nodes represented different head-to-head direct comparisons. The size of the nodes and the thickness of the connection lines respectively represented the sample size of intervention measures and the number of included analysis tests. Statistical heterogeneity was assessed in each comparison using the I^2^ statistic (22). The random-effects model was adopted when I^2^ > 50%; otherwise, the fixed-effects model was adopted (23).

Given the complexity of the analysis, dose differences were ignored. For inconsistent measures, because the included interventions were not looped, loop-based tests could not be applied; instead, a judgment could be made only on the basis of the I2 value within the study. R3.6.1 software was used to complete NMA. For the survival data model, the fixed-effects model based on the combination of hazard ratio (HR) values was adopted. For the binary data, the fixed-effects model based on the combination of RR values was used to evaluate the safety of each treatment measure. To ensure the convergence of the model, the number of iterations was set to 50000, among which the initial number of iterations was 20000. The convergence was evaluated by observing the iteration history diagram. In addition, sequencing results were calculated for each intervention. Based on the surface under the cumulative ranking curve (SUCRA), the cumulative ranking diagram was drawn to illustrate the best treatment. The SUCRA value ranges from 0 to 100, and the closer the value is to 100, the greater the probability that the treatment will be the best treatment. Finally, potential publication bias was tested using Begg’s funnel plots and Egger’s regression test with Stata 15.0 software (StataCorp) (24, 25).

## Results

### Screening and Inclusion of Studies

**Figure 1:**
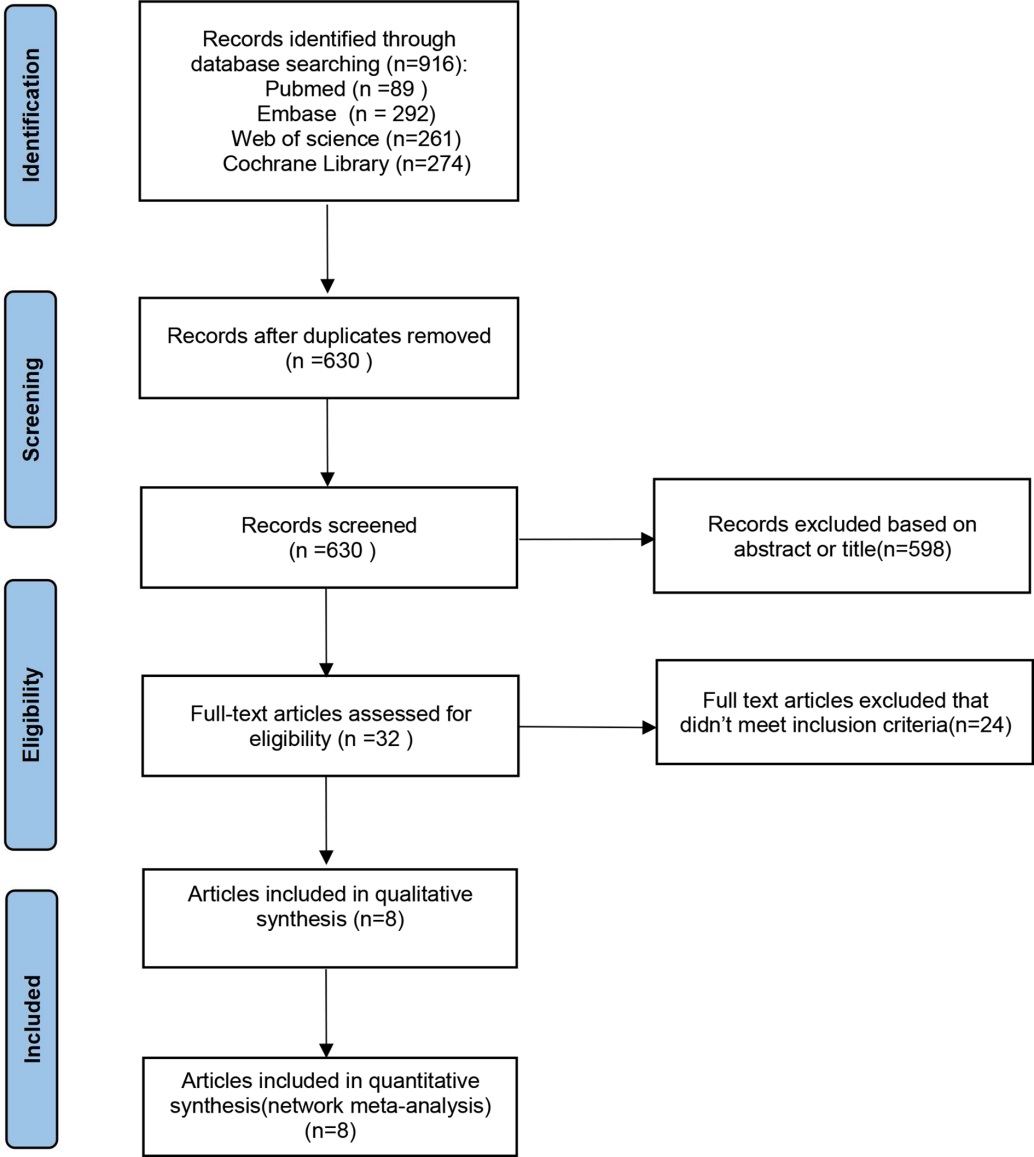
The flow diagram of the study selection process.

**Figure 2:**
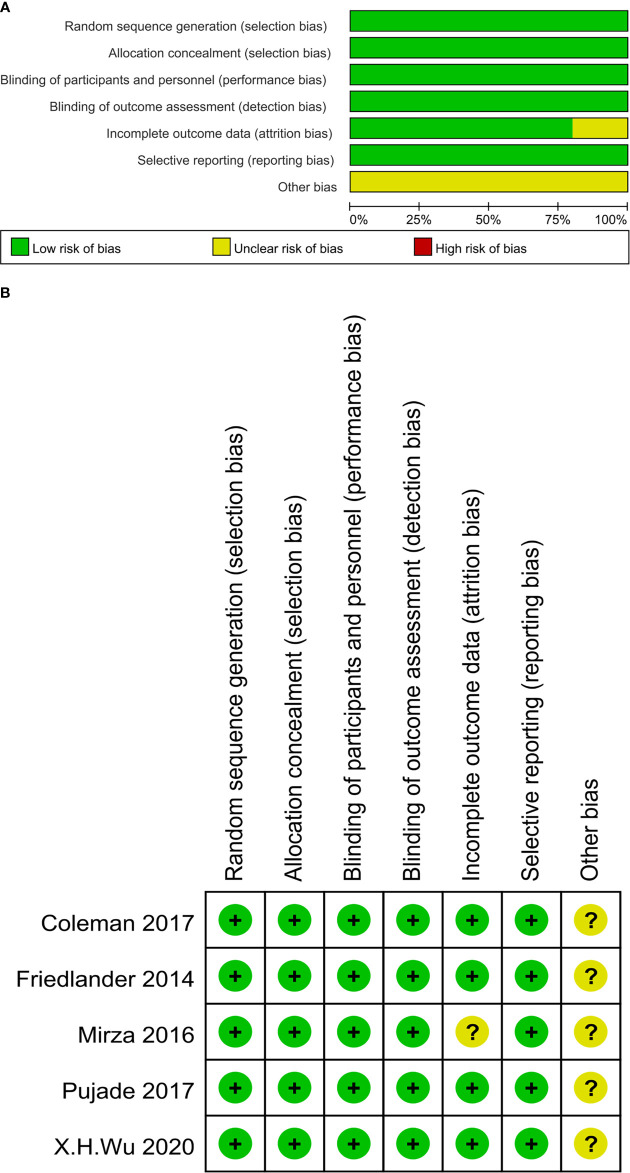
**(A)** The risk of bias graph. The risk judgment for each bias item is presented as the percentage of all included studies. **(B)** The risk of bias summary. The risk judgment for each bias item is presented as the percentage of all included studies.

According to the predetermined search strategy, a total of 916 previous studies were detected from the databases, and the remaining 630 studies were removed by EndNoteX6 software. By reading the titles and abstracts, 598 studies that were obviously inconsistent with the research objects were excluded. For the remaining 32 studies, 24 were excluded by secondary screening according to the exclusion criteria by reading the full text, and a total of eight publications were retained (26–33). These RCTs compared the efficacy and safety of the use of olaparib, niraparib, and rucaparib as maintenance treatment for ROC with those of a placebo. The literature screening process is presented in **Figure 1**. In total, eight publications corresponding to five RCTs published from 2012 to 2021 were included in this analysis, and involved four intervention programs and 1390 patients. The baseline characteristics of the included studies are summarized in **Table 1**. The risk of bias assessment for each study is shown in **Figures 2A**, **B**.

**Table 1:** The basic characteristics of studies included in the meta-analysis.

Register and study abbreviation	Author	Phase	Region	Agents in maintenance phase (dose)	Median age (range) (years)	Number of BRCAm patients	Number of HDR-positive patients	Median PFS (months)
**NCT 00753545**	Ledermann	II	International	Olaprib (N = 136) 400 mg twice daily	58 (21-89)	74	NR	8.4
**STUDY19** (29, 32)				Placebo (N=129)	59 (33-84)	62	NR	4.8
**NCT 01874353**	Pujade- Laurain	III	International	Olaprib (N=196) 300 mg twice daily	56 (51-63)	196	NA	19.1
**SOLO2** (30, 31)				Placebo (N=99)	56 (49-63)	99	NA	5.5
**NCT 01847274**	Mirza	III	International	Niraparib (N=372) 300 mg once daily	57*, 63⁑ (33-84)	138	244	21*, 9.3⁑
**NOVA** (26, 28)				Placebo (N=181)	58*, 61⁑ (34-82)	65	121	5.5*, 3.9⁑
**NCT 03705156**	Wu	III	China	Niraparib (N=177) 300 mg once daily§ 200 mg once daily§	53 (35-78)	65	NA	18.3
**NORA** (27)				Placebo (N=88)	55 (38-72)	35	NA	5.4
**NCT 01968213**	Coleman	III	International	Rucaparib (N=375) 600 mg twice daily	61 (53-67)	130	236	10.8
**ARIEL3** (33)				Placebo (N=189)	62 (53-68)	66	118	5.4

BRCAm, BRCA mutated; PFS, progression-free survival; NR, not reported; NA, not applicable.

*Data related to the gBRCA-mutated population; ⁑Data related to the non-gBRCA-mutated population; § Patients with a bodyweight <77 kg or a platelet count <150×103/μL received 200 mg/day, and all other patients 300 mg/day, as an individualized starting dose (ISD).

**Figure 3:**
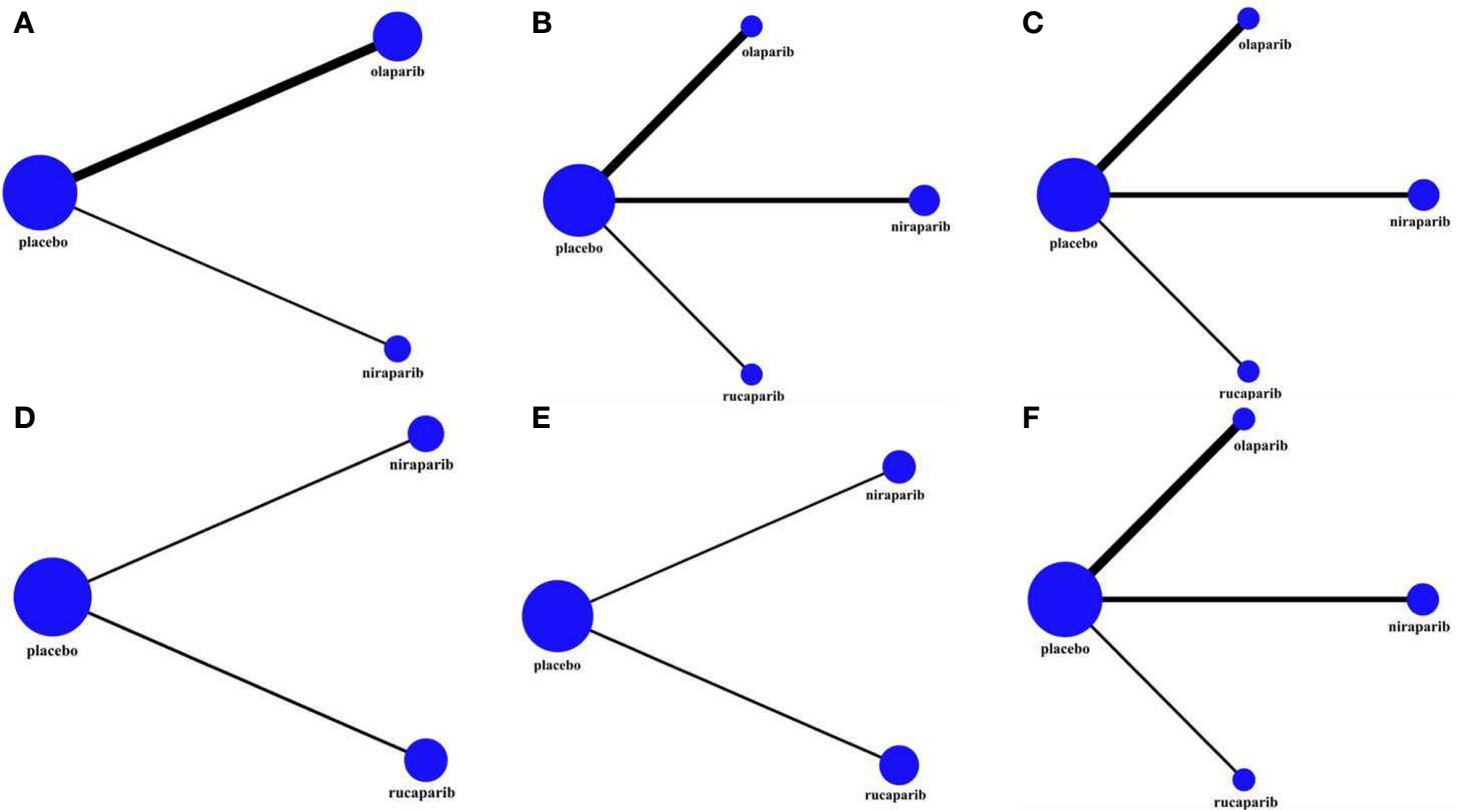
The network plot of the comparisons of all interventions adopted in the network meta-analyses: **(A)** OS in BRCA-mutated patients; **(B)** PFS in the overall population; **(C)** PFS in BRCA-mutated patients; **(D)** PFS in HRD-positive patients; **(E)** PFS in HRD-negative patients; **(F)** AEs of grade 3 to 4 in the maintenance phase. The size of the nodes and the thickness of the connection lines respectively represented the sample size of intervention measures and the number of included analysis tests.

### Network Meta-analysis

#### OS

**Figure 4:**
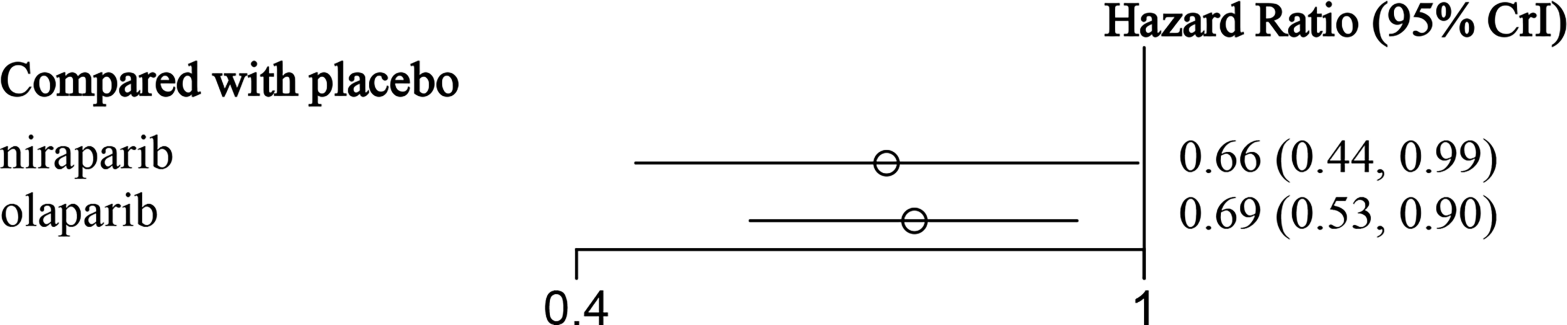
The forest plot of OS in gBRCA-mutated patients.

In this study, three RCTs reported OS data, among which two included an olaparib group (30, 32), and one included a niraparib group (26). As shown in **Table 1**, the follow-up time after the publication of the five research articles included in this paper was not sufficient, so the data related to OS mentioned in this section were re-incorporated into the latest published data of the corresponding research. Because the NOVA trial (26, 28) did not report the OS of the total population, only the gBRCAm population was analyzed (243 in the olaparib group and 138 in the niraparib group), and the related network structure diagrams are displayed in **Figure 3A**. The patients in the placebo group in the SOLO2 and NOVA trials were cross-treated with PARPis in subsequent treatments, which prolonged the OS of the placebo group. Therefore, the data analyzed in this section are the OS results obtained after adjusting for these patients. The results revealed that for patients with gBRCA mutations, niraparib reduced the risk of death by 34% (HR = 0.66, 95% CI: 0.44-0.99), and olaparib reduced the risk of death by 31% (HR = 0.69, 95% CI: 0.53-0.90) (**Figure 4** and **Table 2**). The heterogeneity test showed that there was no significant heterogeneity among the different regimens. According to the SUCRA curve, the PFS ranking probability of the gBRCAm population was as follows: niraparib (77.42%) > olaparib (71.33%) > placebo (1.25%).

**Table 2:** The network meta-analysis of OS in gBRCA-mutated patients.

**Olaparib**	0.96(0.59, 1.55)	1.45(1.11, 1.90)
1.04(0.65, 1.69)	**Niraparib**	1.52(1.01, 2.27)
**0.69(0.53, 0.90)**	**0.66(0.44, 0.99)**	**Placebo**

The data in bold are statistically significant.

#### PFS

**Figure 5:**
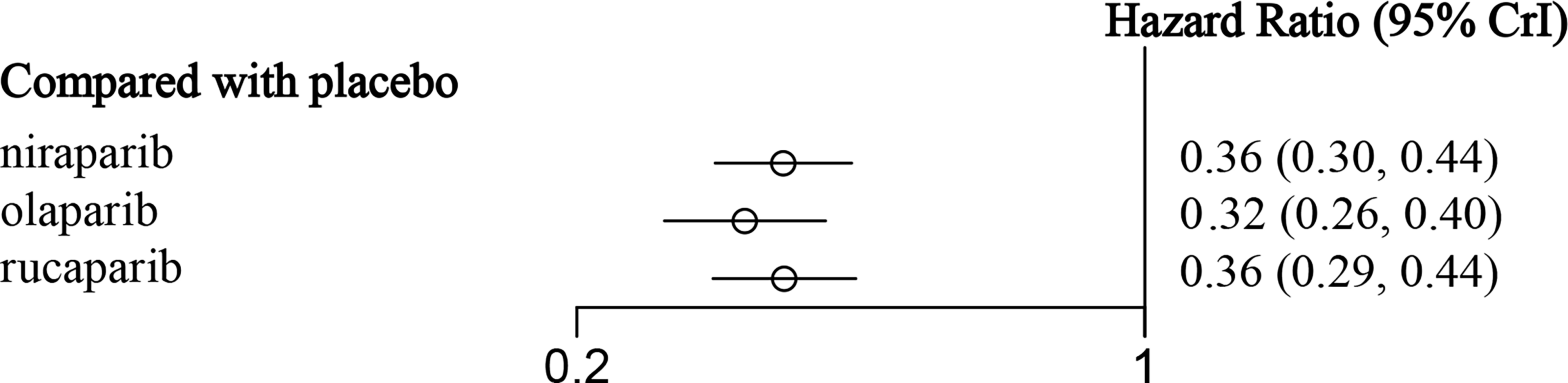
The forest plot of PFS in the overall population.

An NMA of 1390 patients and four interventions in five studies was performed (**Figure 3B**). Among them, 332 patients in two RCTs were treated with olaparib, 549 patients in two RCTs were treated with niraparib, and 375 patients in one RCT were treated with rucaparib. The results showed that in the entire population, olaparib (HR = 0.32, 95% CI: 0.18-0.56), niraparib (HR = 0.35, 95% CI: 0.19-0.61), and rucaparib (HR = 0.36, 95% CI: 0.17-0.82) were very effective in improving PFS as compared with the placebo (**Figure 5** and **Table 3**). There was no significant heterogeneity among the different regimens. According to the SUCRA curve, the PFS ranking probability of the entire population was as follows: olaparib (84.15%) > niraparib (58.16%) > rucaparib (57.69%) > placebo (0%).

**Table 3:** The network meta-analysis of PFS in the overall population.

**Olaparib**	1.11(0.83, 1.50)	1.12(0.82, 1.52)	3.10(2.47, 3.90)
0.90(0.67, 1.21)	**Niraparib**	1.00(0.76, 1.33)	2.78(2.30, 3.38)
0.89(0.66, 1.21)	1.00(0.75, 1.32)	**Rucaparib**	2.78(2.27, 3.40)
**0.32(0.26, 0.40)**	**0.36(0.30, 0.44)**	**0.36(0.29, 0.44)**	**Placebo**

The data in bold are statistically significant.

**Figure 6:**
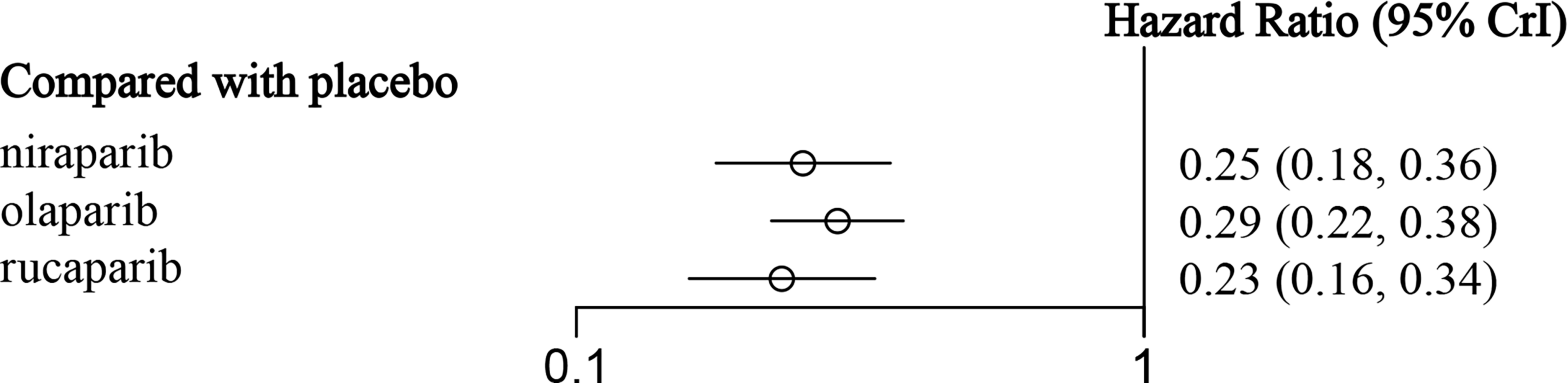
The forest plot of PFS in BRCA-mutated patients.

A corresponding NMA was also performed for the BRCAm subgroup (**Figure 3C**), and the four treatments were compared. All five trials were conducted in the BRCAm population. Among them, 221 patients in two RCTs were treated with olaparib, 202 patients in two RCTs were treated with niraparib, and 130 patients in one RCT were treated with rucaparib. The results demonstrated that for BRCAm ovarian cancer patients, olaparib (HR = 0.29, 95% CI: 0.22-0.38), niraparib (HR = 0.25, 95% CI: 0.18-0.36), and rucaparib (HR = 0.23, 95% CI: 0.16-0.34) were very effective in improving PFS as compared with the placebo (**Figure 6** and **Table 4**). There was no significant heterogeneity among the different regimens. According to the SUCRA curve, the PFS ranking probability of BRCAm patients was as follows: rucaparib (82.04%) > niraparib (69.96%) > olaparib (48.00%) > placebo (0%).

**Table 4:** The network meta-analysis of PFS in BRCA-mutated patients.

**Olaparib**	0.87(0.56, 1.36)	0.80(0.50, 1.27)	3.47(2.66, 4.54)
1.15(0.74, 1.79)	**Niraparib**	0.92(0.55, 1.54)	3.98(2.80, 5.67)
1.25(0.79, 1.99)	1.09(0.65, 1.83)	**Rucaparib**	4.35(2.98, 6.34)
**0.29(0.22, 0.38)**	**0.25(0.18, 0.36)**	**0.23(0.16, 0.34)**	**Placebo**

The data in bold are statistically significant.

**Figure 7:**
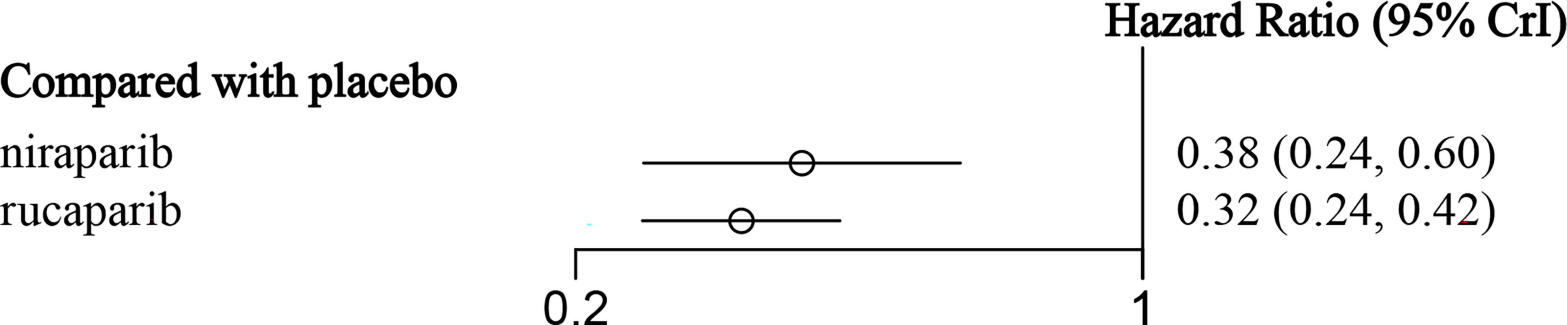
The forest plot of PFS in HRD-positive patients.

**Figure 8:**
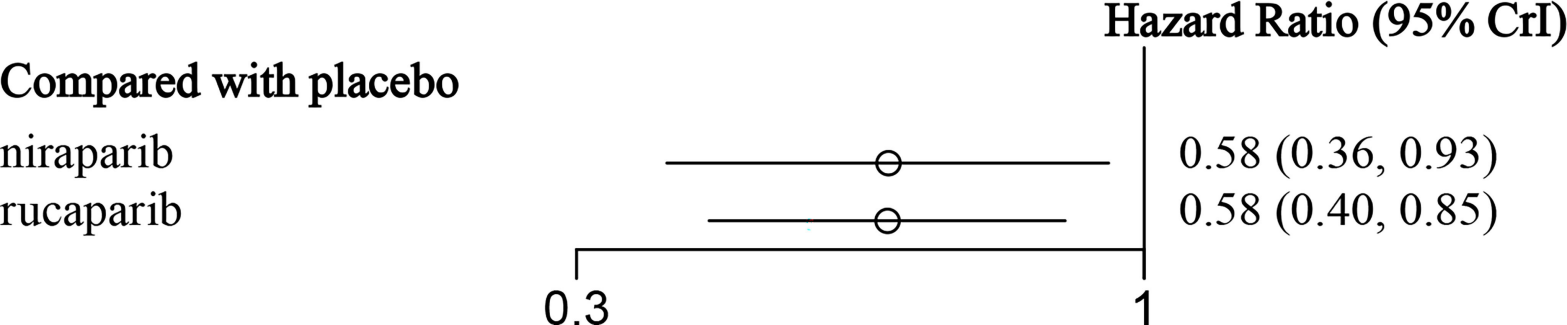
The forest plot of PFS in HRD-negative patients.

In the subgroup of HRD-positive patients, only the NOVA and ARIEL3 studies reported relevant data (**Figures 3D, E** **)** for niraparib and rucaparib, respectively. The results showed that niraparib (HR = 0.38, 95% CI: 0.24-0.60) and rucaparib (HR = 0.32, 95% CI: 0.24-0.43) were both very effective in HRD-positive patients as compared with the placebo (**Figure 7** and **Table 5**). Similarly, for HRD-negative patients, niraparib (HR = 0.58, 95% CI: 0.36-0.93) and rucaparib (HR = 0.58, 95% CI: 0.40-0.85) were found to prolong PFS (**Figure 8** and **Table 6**). There was no significant heterogeneity among the different regimens. According to the SUCRA curve, the PFS ranking probability of HRD-positive patients was as follows: rucaparib (86.82%) > niraparib (63.18%) > placebo (0%); that of HRD-negative patients was as follows: rucaparib (74.93%) > niraparib (74.36%) > placebo (0.71%).

**Table 5:** The network meta-analysis of PFS in HRD-positive patients.

**Niraparib**	0.84(0.50, 1.43)	2.63(1.68, 4.12)
1.19(0.70, 2.02)	**Rucaparib**	3.13(2.36, 4.14)
**0.38(0.24, 0.60)**	**0.32(0.24, 0.42)**	**Placebo**

The data in bold are statistically significant.

**Table 6:** The network meta-analysis of PFS in HRD-negative patients.

**Niraparib**	1.00(0.55, 1.83)	1.72(1.08, 2.75)
1.00(0.55, 1.83)	**Rucaparib**	1.72(1.18, 2.52)
**0.58(0.36, 0.93)**	**0.58(0.40, 0.85)**	**Placebo**

The data in bold are statistically significant.

### Adverse Effects

**Figure 9:**
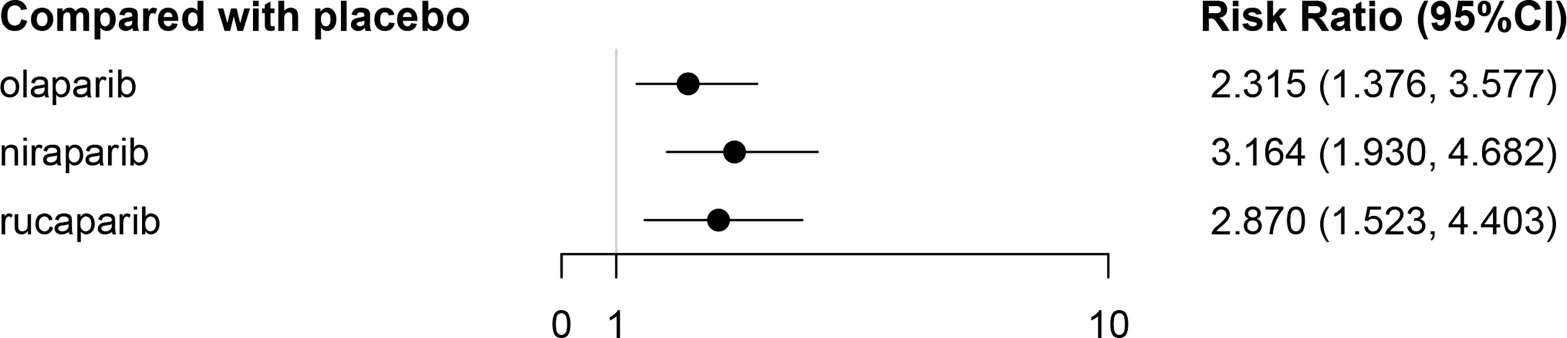
The forest plot of grade 3-4 AEs in the maintenance phase.

As patients with grade 1-2 adverse effects (AEs) can still tolerate treatment and may not require special treatment, the AEs considered in this study were grade 3-4, and the results are reflected by the RR value and the 95% CI. **Figure 3F** exhibits the corresponding network diagram. The results revealed that, compared with the placebo, olaparib (RR = 2.31, 95% CI: 1.38-3.58), niraparib (RR = 3.16, 95% CI: 1.93–4.68), and rucaparib (RR = 2.87, 95% CI: 1.52-4.40) can lead to a higher risk of grade 3-4 AEs (**Figure 9** and **Table 7**). All evaluated PARPi regimens were found to significantly increase the number of grade 3-4 AEs in ROC patients as compared to the placebo. The heterogeneity test showed that there was no significant heterogeneity among the different regimens. According to the SUCRA curve, the ranking probability of grade 3-4 AEs was as follows: placebo (99.75%) > olaparib (59.00%) > rucaparib (29.45%) > niraparib (11.80%).

**Table 7:** The network meta-analysis of grade 3-4 AEs in the maintenance phase.

**Olaparib**	1.37(0.88, 2.11)	1.24(0.69, 1.97)	0.43(0.28, 0.73)
0.73(0.47, 1.13)	**Niraparib**	0.91(0.53, 1.34)	0.32(0.21, 0.52)
0.81(0.51, 1.44)	1.10(0.74, 1.88)	**Rucaparib**	0.35(0.23, 0.66)
**2.32(1.38, 3.58)**	**3.16(1.93, 4.68)**	**2.87(1.52, 4.40)**	**Placebo**

**Figure 10:**
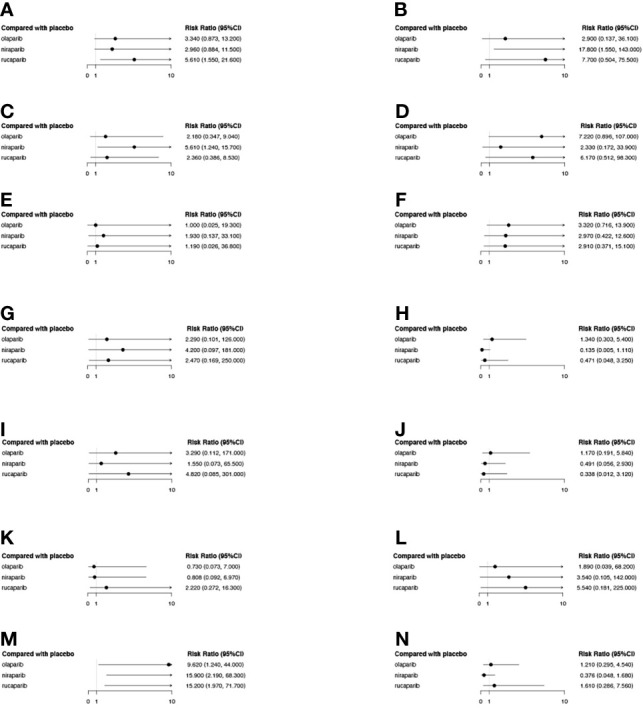
The forest plot of the relative risk (RR) of grade 3-4 AEs of different PARPis as compared to the placebo. Each subplot represents a different AE. **(A)** Vomiting; **(B)** Thrombocytopenia; **(C)** Neutropenia; **(D)** Nausea; **(E)** Headache; **(F)** Fatigue or asthenia; **(G)** Dizziness; **(H)** Diarrhea; **(I)** Decreased appetite; **(J)** Cough; **(K)** Constipation; **(L)** Back pain; **(M)** Anemia; **(N)** Abdominal pain.

Specific analyses were also conducted for the co-occurrence of grade 3-4 AEs reported for the three drugs (**Figure 10**), and the results revealed that, in terms of hematological toxicity, all three PARPi treatments caused anemia as compared to the placebo. Moreover, the incidence of thrombocytopenia and neutropenia in the niraparib group was significantly higher than that in the olaparib and rucaparib groups. Furthermore, rucaparib was found to be associated with a higher rate of grade 3-4 vomiting than olaparib and niraparib. However, there was no statistical difference among the three PARPis in terms of grade 3-4 other non-hematological toxicity, such as nausea, decreased appetite, diarrhea, constipation, cough, dizziness, headache, fatigue or asthenia, abdominal pain, and back pain.

### Publication Bias

**Figure 11:**
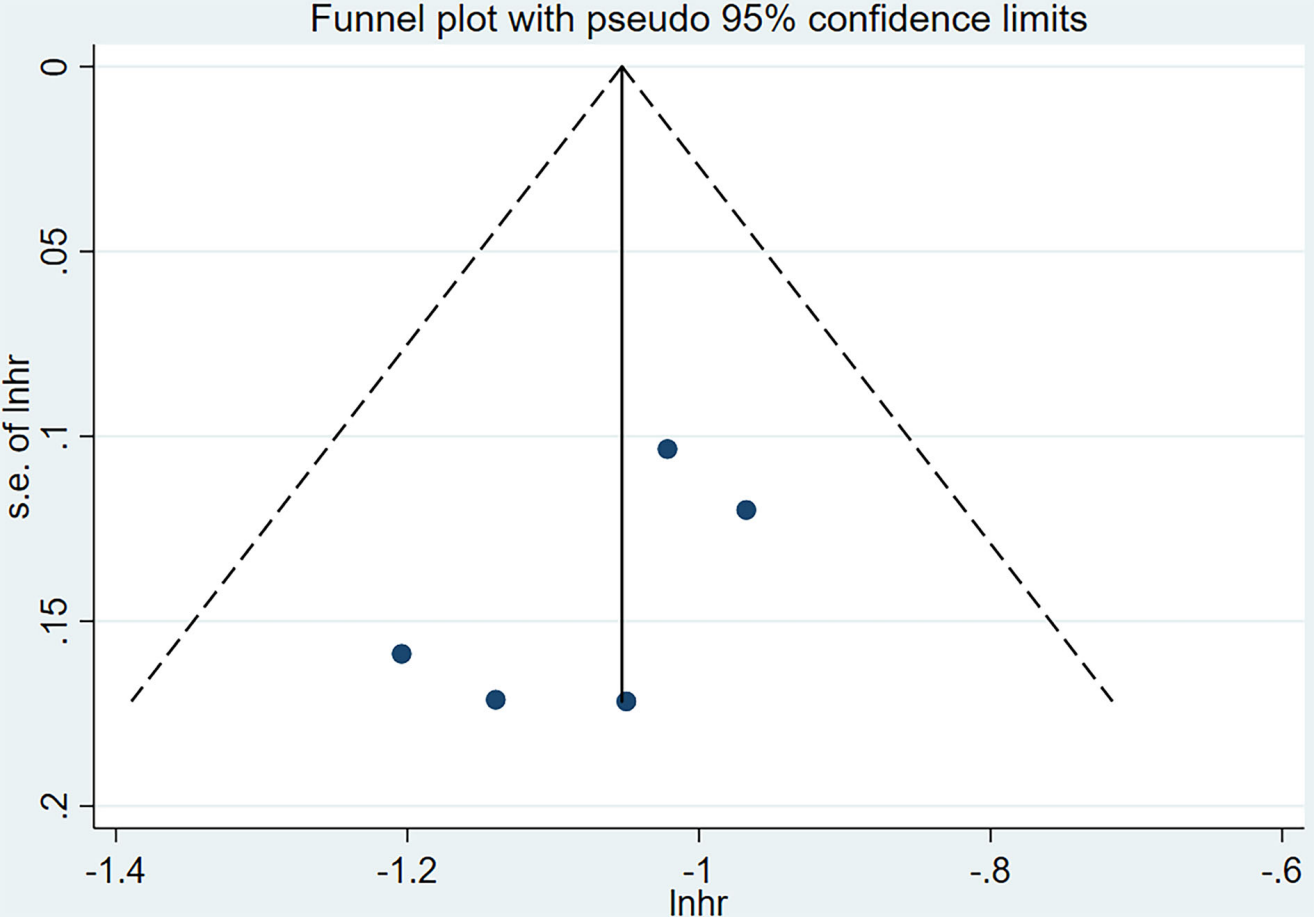
Funnel plots for publication bias.

Visual inspection of the Begg funnel plot did not identify substantial asymmetry (**Figure 11**). Publication bias was examined by using Egger’s (P = 0.806) and Begg’s (P = 0.201) tests, and the results indicate that there was no publication bias.

## Discussion

This is the first direct comparison of the long-term efficacy of PARPi monotherapy for patients with ROC in terms of prolonging OS. Many clinical studies have found that the benefit of PFS does not translate into the benefit of OS, which is the gold standard of tumor therapy assessment (18, 19). Although Shao et al. (16) analyzed OS, they combined newly diagnosed ovarian cancer with ROC, which undoubtedly increased the heterogeneity of the study. Moreover, the OS data were not mature at that time, so the final analysis results lacked certain guiding significance. Because OS itself is affected by a variety of factors, such as a large amount of missing data and a high crossover rate, after analyzing the data adjusted by the inverse probability weighting method, it was found that maintenance treatment with niraparib and olaparib tended to improve OS in the gBRCA-mutated population. In addition, although the OS of the general population was not analyzed, Friedlander’s updated data on STUDY19 (29, 32) demonstrated that patients receiving olaparib maintenance treatment had a better OS than those receiving the placebo (HR = 0.73, 95% CI: 0.55-0.96, P = 0.025), although no nodes of statistical significance were reached (P < 0.0095). In the final SOLO2 data reported by Poveda in 2021 (30), the median OS in the olaparib group was found to be 51.7 months as compared with 31.8 months in the placebo group, and olaparib reduced the risk of death by 26% (HR = 0.74, 95% CI: 0.54-1.00). However, in this study, 38.4% of patients in the placebo group were cross-treated with olaparib during subsequent treatment, resulting in prolonged OS in the placebo group. After adjusting for these patients, the median OS in the placebo group was found to be 35.4 months, and olaparib was found to prolong OS by 16.3 months and decrease the risk of mortality by 44% (HR = 0.56, 95% CI: 0.35-0.97). The release of OS data from these two studies, especially the SOLO2 data, demonstrates that maintenance therapy can significantly prolong OS, and can further extend OS as compared to the previous emphasis on the extension of PFS. Since then, the maintenance treatment of ovarian cancer has officially entered the prolonged OS era.

The three PARPis used as monotherapy maintenance therapy for ROC were found to be highly effective in improving PFS as compared with the placebo, and there was no statistical difference between the total population and the BRCAm population, which is consistent with the results of Xu et al. (34). This provides a basis and the possibility of the expanded use of PARPis to the entire ROC patient population, as does the fact that niraparib and rucaparib can benefit PFS in people with HRD mutations, regardless of positive or negative HRD status. In terms of AEs, a higher incidence of AEs was found in patients treated with PARPis. However, unlike previously published studies (35, 36), the current reanalysis of the newly updated data from STUDY19 and SOLO2 revealed that the safety of olaparib was not superior to that of niraparib and rucaparib. However, this analysis showed that the incidence of thrombocytopenia and neutropenia caused by niraparib was higher than that caused by the other two PARPis. Differences in toxicity distribution are of clinical importance and may influence treatment decisions. In a retrospective study of the NOVA trial, it was found that the treatment of most patients taking niraparib at a starting dose of 300 mg was interrupted or reduced due to specific hematological and non-hematological toxicity (35). After dose adjustment (including 300, 200, and 100 mg), most patients achieved stability. Researchers evaluated the PFS of the remaining patients, and found that the PFS of the patients for whom the dose was reduced to 200 or 100 mg was consistent with that of the patients who maintained the 300-mg starting dose. Moreover, once the patient reached the optimal individualized dose, the efficacy was not found to be affected. Furthermore, a corresponding reduction in AEs was observed after the dose adjustment of niraparib, especially thrombocytopenia. This also suggests that the AEs associated with niraparib can be reduced by reducing the dosage without reducing efficacy. Although this retrospective study appears to show that the AEs are dose-dependent and that lowering the dose does not affect the PFS, these findings have yet to be confirmed in an RCT. Moreover, although the probability of grade 3-4 AEs caused by PARPis was found to be higher than that in the placebo group, the three PARPis exhibited no statistical difference in various non-hematological toxicities except vomiting, and AEs were generally controlled. The management of AEs included supportive care and dose modifications (including treatment interruption or dose reduction) (28, 31, 33).

The difference between these three drugs may be attributed to their selectivity; one study of 10 clinical PARPis demonstrated that niraparib was more selective for PARP1 and PARP2, while olaparib and rucaparib were more potent inhibitors of PARP1 but less selective (36). Previous studies have concluded that olaparib and rucaparib have different *in vitro* affinity for a set of different kinases; rucaparib was found to inhibit at least nine kinases with micromolar affinity, while olaparib was found to be inactive for all 16 tested kinases, and was more selective (37). In addition, another study found that the binding affinities of these three drugs were different. For example, rucaparib was found to inhibit CDK16, PIM3, and DYRK1A/B, while niraparib was found to inhibit only DYRK1A/B (38). The pharmacokinetics of the three drugs are also different. Niraparib is metabolized by carboxylesterase-catalyzed amide hydrolysis in the liver with an average half-life of 36 h, while olaparib and rucaparib are metabolized mainly through the cytochrome P450 enzyme pathway with average half-lives of 14 and 17 h, respectively (39). In addition, Yu et al. used a series of related PARPis with different PARP1 capture capabilities, as well as “non-captured” PARPPROTAC, to prove that although PARPis can effectively block PARP1 enzyme activity, there are different levels of PARP1 trapping (niraparib > rucaparib > olaparib); moreover, it was found that the activation of the innate immune response to the anti-tumor effect and the corresponding cytotoxicity levels are primarily dependent on PARP1 trapping, and the correlations are positive (40–43). These findings may help explain the differences in the efficacy and toxicity of PARPis.

A total of five RCTs were included in this NMA, and were of high quality and published in world-renowned medical journals and magazines. However, the number of included trials was small and requires further supplementation. Although the existing literature was searched in strict accordance with the inclusion and exclusion criteria, there remained a certain probability of omission, especially if some trials had not been publicly published, or if the articles were not published in Chinese or English, which may have also caused publication bias. Second, both phase II and phase III trials were included in the current study, which may be a potential source of bias. Finally, the research sample size included in this study was small, the clinical observation indicators of some studies were not comprehensive, and the follow-up time was short, which may have had a certain impact on the research results.

Despite these limitations, this research is characterized by the following key advantages. First, this is the first NMA to compare the efficacy and safety of PARPi monotherapy for platinum-sensitive ROC. Second, only studies that investigated platinum-sensitive ROC patients were included, and the baseline characteristics of all trials were similar. In addition, the population was also stratified into the BRCAm population, HRD-positive patients, HRD-negative patients, and the entire population. The stratification results allowed for the better evaluation of the efficacy of PARPis in different HRD phenotypes, which provides the possibility to expand the use of PARPis to the entire population. Furthermore, the NORA trial (NCT03705156) (26) included in this article was the first large-scale randomized controlled phase III clinical study of PARPi treatment in ROC patients in China. It prospectively validates the efficacy and safety of an individualized starting dose of niraparib for maintenance therapy in platinum-sensitive ROC patients, thereby providing more convincing data for Asian ovarian cancer patients. Most importantly, this study was also the first to perform an OS analysis of PARPis, which most intuitively reflects clinical benefits and provides a basis for further establishing PARPis as a standard of maintenance therapy after surgery or chemotherapy.

In addition to the three PARPis considered in this work, domestic PARPis such as fluzoparib and pamiparib have also become a new force to be reckoned with. On December 14, 2020, the first domestic PARPi, fluzoparib, which was developed by Hengrui, was officially approved for marketing by the National Medical Products Administration (NMPA) (44). Although the data of its phase III trial FZPL-III-301-OC (NCT03863860) has not yet been published, the median data indicate that the PFS of the experimental group was 12.9 months, as compared with 5.5 months in the placebo group (HR = 0.25, 95% CI: 0.17-0.36, P < 0.0001), and it reduced the risk of disease progression and death by 75%. Regardless of whether gBRCA1/2 was mutated, all subgroups of patients were found to benefit from fluzoparib treatment. In addition, pamiparib, which was developed by BeiGene, was also formally approved on May 7, 2021, for the treatment of gBRCA-mutated patients with ROC, fallopian tube cancer, or primary peritoneal cancer who have undergone second-line or higher chemotherapy. The results of the phase I/II BGB-290-AU-002 study (NCT03333915) showed that the objective response rate (ORR) for platinum-sensitive ROC patients was 64.6% (95% CI: 53.3%-74.9%), and the median PFS was 15.2 months (10.35, NE). For platinum-resistant ROC patients, the ORR was 31.6% (95% CI: 12.6%-56.6%), and the median PFS was 6.2 months (4.11, NE) (45). The phase III BGB-290-302 trial of pamiparib is currently in progress, and the report of the final data is expected to change the pattern of ovarian cancer treatment and open up a new era of “de-chemotherapy” treatment.

Since the end of 2019, the COVID-19 pandemic has greatly changed our lives and gradually shifted the medical and health system to telemedicine. Cancer patients are more likely to be infected with COVID-19 than non-cancer patients due to the systemic immunosuppression caused by malignant tumors and anti-cancer treatments, such as chemotherapy and surgery (6, 7). Studies have shown that ACE2, the receptor *via* which COVID-19 invades cells, is highly expressed in a variety of tumors, in which genetic abnormalities such as ACE2 mutations and abnormal copy number amplification can occur. Tumors with a high expression of ACE2 may be more susceptible to COVID-19 (46, 47). Moreover, repeated hospital visits are also a potential risk factor for COVID-19 infection due to low autoimmunity. Therefore, experts have pointed out (8) that under the premise of pandemic prevention, after the risk of pneumonia in patients is excluded, patients should be reasonably evaluated and stratified. The principle of treatment is to use convenient, safe, and effective treatments. Priority should be given to relatively mild and convenient oral drugs, or to safe drugs with less adverse reactions and few sequelae effects (such as endocrine, targeted, or immunotherapy drugs) to reduce leukocyte decrease and fever.

In addition, cancer patients are prone to different degrees of anxiety, depression, and other mental health problems. During the pandemic period, the established diagnosis and treatment plan and time of patients are affected, which is more likely to increase their mental burden. Therefore, in the process of diagnosis and treatment communication, clinicians can evaluate the mental state of patients, and thoroughly explain and communicate their condition. Furthermore, home care also plays a very important role in the holistic treatment of patients; it can provide patients with daily care and emotional and spiritual support, and can sometimes even act as the largest pillar and motivation for patients.

In conclusion, both olaparib and niraparib maintenance therapy were found to significantly prolong OS in patients with gBRCA mutations, and olaparib exhibited a definite effect in prolonging OS in the general population. Monotherapy with all three PARPis can prolong PFS with no significant difference in efficacy. In terms of safety, the use of PARPis has been associated with AEs, which are generally controllable. Home oral PARPi therapy can balance tumor treatment and pandemic prevention and control, and can realize disease control for ovarian cancer patients. Against the background of the current COVID-19 pandemic, PARPi treatment is the most convenient, safe, and effective home-based treatment for patients with ROC.

## Data Availability Statement

The original contributions presented in this study are included in the article/supplementary material. Further inquiries can be directed to the corresponding authors.

## Author Contributions

HW, ZH, and MW contributed to the conception and design of the study. YZ, YG, and BJ collected and assessed the literature. WM, HL, and HZ performed the statistical analysis. HW and MW wrote the first draft of the manuscript; All authors contributed to the article and approved the submitted version.

## Conflict of Interest

The authors declare that the research was conducted in the absence of any commercial or financial relationships that could be construed as a potential conflict of interest.

## Publisher’s Note

All claims expressed in this article are solely those of the authors and do not necessarily represent those of their affiliated organizations, or those of the publisher, the editors and the reviewers. Any product that may be evaluated in this article, or claim that may be made by its manufacturer, is not guaranteed or endorsed by the publisher.
